# Implementing and evaluating shared decision-making before transcatheter aortic valve implantation with a dedicated pathway and questionnaire

**DOI:** 10.1093/ehjopen/oeae095

**Published:** 2024-11-04

**Authors:** Ermes Carulli, Suzy Browne, Sara Woolley, Alexander Tindale, Alison Pottle, Kate Nagle, Rebecca Lane, Navin Chandra, Niket Patel, Rodney De Palma, Gareth Barnes, Tito Kabir, Vasileios Panoulas, David Smith, Robert Smith, Sharon Clernon, Ee Ling Heng, Mohammed Akhtar, Mark Bowers, Ian McGovern, Thomas Lüscher, Miles Dalby

**Affiliations:** Guy's and St Thomas' NHS Foundation Trust, Harefield Hospital, Department of Cardiology, Hill End Rd, Harefield, Uxbridge UB9 6JH, UK; Doctoral School in Translational Medicine, University of Milan, Via Festa del Perdono 7, 20122 Milan, Italy; Guy's and St Thomas' NHS Foundation Trust, Harefield Hospital, Department of Cardiology, Hill End Rd, Harefield, Uxbridge UB9 6JH, UK; Guy's and St Thomas' NHS Foundation Trust, Harefield Hospital, Department of Cardiology, Hill End Rd, Harefield, Uxbridge UB9 6JH, UK; Guy's and St Thomas' NHS Foundation Trust, Harefield Hospital, Department of Cardiology, Hill End Rd, Harefield, Uxbridge UB9 6JH, UK; Imperial College London, Exhibition Road, London SW7 2AZ, UK; Guy's and St Thomas' NHS Foundation Trust, Harefield Hospital, Department of Cardiology, Hill End Rd, Harefield, Uxbridge UB9 6JH, UK; Guy's and St Thomas' NHS Foundation Trust, Harefield Hospital, Department of Cardiology, Hill End Rd, Harefield, Uxbridge UB9 6JH, UK; Guy's and St Thomas' NHS Foundation Trust, Harefield Hospital, Department of Cardiology, Hill End Rd, Harefield, Uxbridge UB9 6JH, UK; Guy's and St Thomas' NHS Foundation Trust, Harefield Hospital, Department of Cardiology, Hill End Rd, Harefield, Uxbridge UB9 6JH, UK; Frimley Health NHS Foundation Trust, Frimley Park Hospital, Portsmouth Road, Frimley GU16 7U, UK; Royal Free London NHS Foundation Trust, Pond St, London NW3 2QG, UK; Buckinghamshire Healthcare NHS Trust, Stoke Mandeville Hospital, Mandeville Road, Buckinghamshire, Aylesbury HP21 8AL, UK; Ashford and St Peter's Hospitals NHS Foundation Trust, St Peters Hospital, Guildford Road, Chertsey KT16 0PZ, UK; Guy's and St Thomas' NHS Foundation Trust, Harefield Hospital, Department of Cardiology, Hill End Rd, Harefield, Uxbridge UB9 6JH, UK; Guy's and St Thomas' NHS Foundation Trust, Harefield Hospital, Department of Cardiology, Hill End Rd, Harefield, Uxbridge UB9 6JH, UK; Imperial College London, Exhibition Road, London SW7 2AZ, UK; Guy's and St Thomas' NHS Foundation Trust, Harefield Hospital, Department of Cardiology, Hill End Rd, Harefield, Uxbridge UB9 6JH, UK; Guy's and St Thomas' NHS Foundation Trust, Harefield Hospital, Department of Cardiology, Hill End Rd, Harefield, Uxbridge UB9 6JH, UK; Guy's and St Thomas' NHS Foundation Trust, Harefield Hospital, Department of Cardiology, Hill End Rd, Harefield, Uxbridge UB9 6JH, UK; Guy's and St Thomas' NHS Foundation Trust, Harefield Hospital, Department of Cardiology, Hill End Rd, Harefield, Uxbridge UB9 6JH, UK; Guy's and St Thomas' NHS Foundation Trust, Harefield Hospital, Department of Cardiology, Hill End Rd, Harefield, Uxbridge UB9 6JH, UK; Guy's and St Thomas' NHS Foundation Trust, Harefield Hospital, Department of Cardiology, Hill End Rd, Harefield, Uxbridge UB9 6JH, UK; Guy's and St Thomas' NHS Foundation Trust, Harefield Hospital, Department of Cardiology, Hill End Rd, Harefield, Uxbridge UB9 6JH, UK; Guy's and St Thomas' NHS Foundation Trust, Harefield Hospital, Department of Cardiology, Hill End Rd, Harefield, Uxbridge UB9 6JH, UK; Imperial College London, Exhibition Road, London SW7 2AZ, UK; Guy's and St Thomas' NHS Foundation Trust, Harefield Hospital, Department of Cardiology, Hill End Rd, Harefield, Uxbridge UB9 6JH, UK; Imperial College London, Exhibition Road, London SW7 2AZ, UK

**Keywords:** Shared decision-making, Transcatheter aortic valve implantation, Digital health, Patient engagement

## Abstract

**Aims:**

Transcatheter aortic valve implantation (TAVI) is an alternative to surgical aortic valve replacement for patients with aortic valve stenosis. The choice between TAVI, surgery, or a conservative approach should be based upon multiple factors including clinical considerations, technical feasibility, and informed patient preference. In this context, engaging patients in a shared decision-making (SDM) process becomes essential, but this practice is generally underused.

**Methods and results:**

To comply with the European and UK national guidelines, in January 2023 we established a structured SDM pathway in which patients are offered virtual/physical decision aids and after 1 week are invited to a meeting to reach a shared decision. From December 2022 to June 2023, a custom-developed questionnaire was prospectively administered to 23 patients prior to, and 38 patients after, the implementation of the SDM pathway. The answers to 12 core questions were recorded on a Likert scale (1–5). Global satisfaction, as measured by mean Likert score, was significantly higher for the post-SDM group than for the pre-SDM group (4.46 ± 0.14 vs. 3.78 ± 0.30, *P* < 0.001). The percentage of positive (Likert 4–5) responses was significantly higher in the post-SDM group (289/312, 92.6% vs. 155/234, 66.2%, *P* < 0.001). The percentage of negative (Likert 1–2) responses was significantly lower in the post-SDM group (5/312, 1.6% vs. 53/234, 22.6%, *P* < 0.001).

**Conclusion:**

The SDM pathway proved effective in delivering SDM in compliance with national and international guidance. A similar approach leveraging digital technology to minimize cost and enhance patient convenience could be implemented for other treatments and across other institutions.

## Graphical abstract

**Graphical Abstract:**
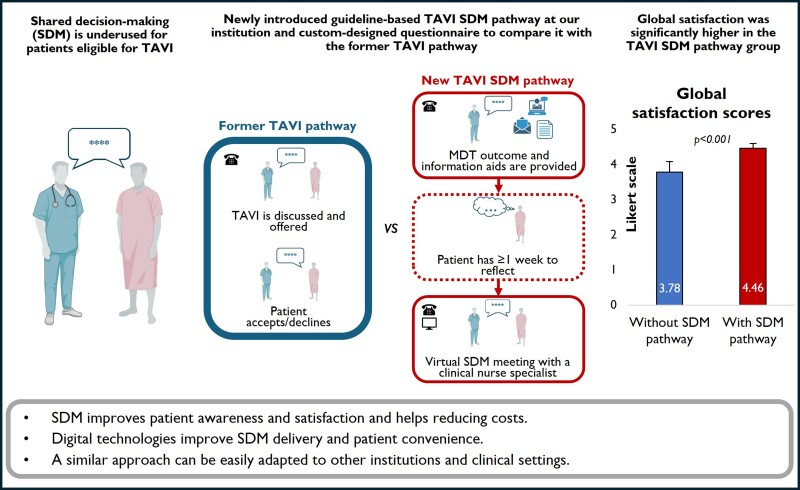

## Introduction

### The underuse of shared decision-making in routine clinical practice

Transcatheter aortic valve implantation (TAVI) was first performed in humans in 2002.^1^ Since then, its implementation has gradually increased as an alternative to surgery for aortic valve stenosis patients at extreme surgical risk to high, intermediate, and now selected patients at low surgical risk.^2–4^ However, TAVI is still a major intervention with serious potential adverse events, including mortality, stroke, vascular complication, complete heart block/need for pacemaker implantation, and paravalvular regurgitation.^4,5^ Moreover, only biological valves (therefore excluding mechanical options) can be implanted, with limited long-term durability mandating consideration of future repeat procedures.^6,7^ In addition, consideration needs to be given to potential problems with coronary artery access for percutaneous procedures after TAVI and in particular after future potential ‘valve in valve’.

**Figure 1:**
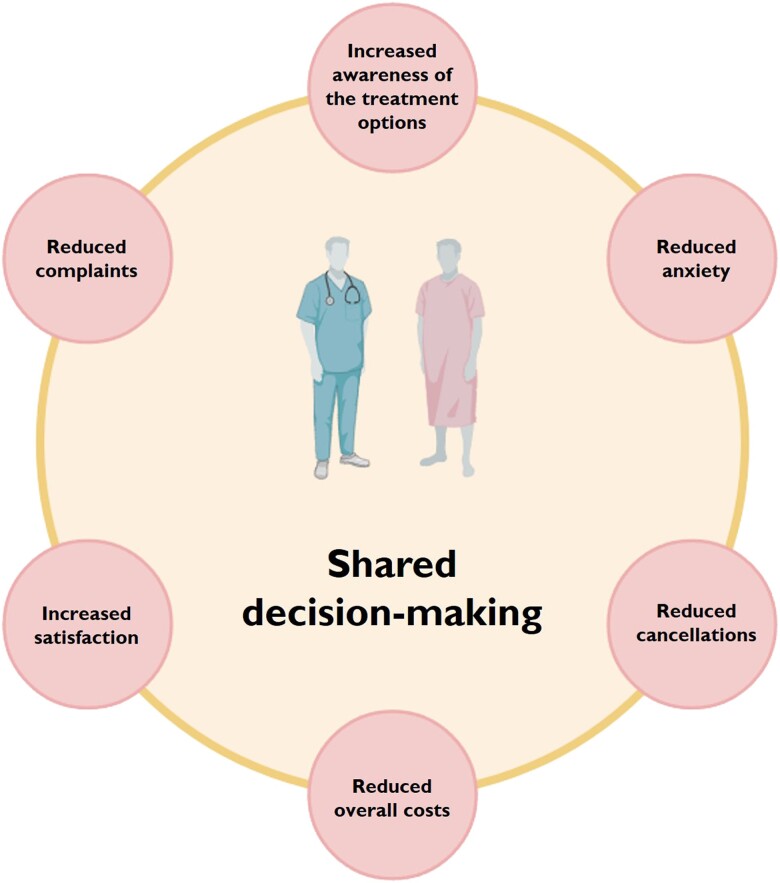
Advantages of the shared decision-making process. This figure summarizes the main benefits of shared decision-making based on the available evidence. These benefits apply for every field of medicine but are particularly relevant for transcatheter aortic valve implantation, where the treatment options also include surgical replacement and medical management, and for the final decision, the patient’s preferences are particularly important. Image created with the help of BioRender.com.

Current European Society of Cardiology/European Association for Cardio-Thoracic Surgery guidelines (2021) recommend that the choice between TAVI and surgery be based on multiple factors including age (with a generally accepted cut-off of 75 years), life expectancy, frailty, anatomy, and local experience and also taking into consideration the durability issue of biological valves. Notably, guidelines highlight that these factors should be discussed with the patient and family to allow an informed treatment choice.^4^ In fact, especially in cases where the treatment options are so different in terms of invasiveness, potential need for re-intervention, and possible complications, a shared decision-making (SDM) process between the clinical team and the patient becomes of paramount importance.^8^ Based on the National Institute of Health and Care Excellence (NICE) guidelines,^9^ SDM is a collaborative process empowering patients and healthcare professionals to engage in discussion around treatment goals and options. It involves selection of treatment based on both evidence and the patient’s preferences and ensures that the patient understands the risks, benefits, and possible consequences of the different therapeutic alternatives. This process offers a better alignment with the patient’s values and needs, less anxiety, reduction in unwarranted variation in care and costs, and ultimately improved patient satisfaction and health outcomes (*Figure 1*).^10,11^ The benefits of active patient involvement are further highlighted by international patient organizations such as the Global Heart Hub, which provides a guidance for patients with heart valve disease to help them navigate the SDM process.^12^

Nevertheless, the application of SDM is often limited in real-world clinical practice. Based on the experience from the ‘Making Good Decisions in Collaboration’ programme commissioned in the UK in 2010, the main obstacles to SDM perceived by clinicians included the lack of time due to other demands and priorities, the lack of standardized tools to implement and measure SDM, and the false impression that the patients generally prefer not to be engaged in SDM or that SDM is already being performed.^8^

Several tools to measure SDM are described in the literature and differ according to the perspective they are based on. From a patient perspective, examples are SDM Questionnaire-9 (SDM-Q-9),^13^ Combined Outcome Measure for Risk Communication and Treatment Decision-Making Effectiveness,^14^ or Perceived Involvement in Care Scale.^15^ From a clinician perspective, an example is SDM-Q-Physician Version.^16^ From an observer perspective, an example is Decision Support Analysis Tool^17^ or Observing Patient Involvement in Decision-Making (OPTION).^18^ However, there is no consensus on the most appropriate tool, and their validity is generally not formally established; therefore, there is no gold standard to date.^19^

### Experience with a shared decision-making pathway for transcatheter aortic valve implantation

To implement formal SDM in our practice and measure its impact on patient understanding of the treatment options and general satisfaction, we recently designed a structured SDM pathway and systematically incorporated it into our TAVI pathway in 2023. Importantly, we aimed to incorporate SDM with limited resource and no extra staff.

The SDM pathway was structured to address the NICE recommendations, which represent the reference guidelines in the UK, and to comply with the Professional Record Standards Body (PRSB) indications, which are in turn informed by NICE guidelines to provide a framework for optimal medical practice.^20^ We anticipated that this would significantly enhance patient engagement in SDM.

**Figure 2:**
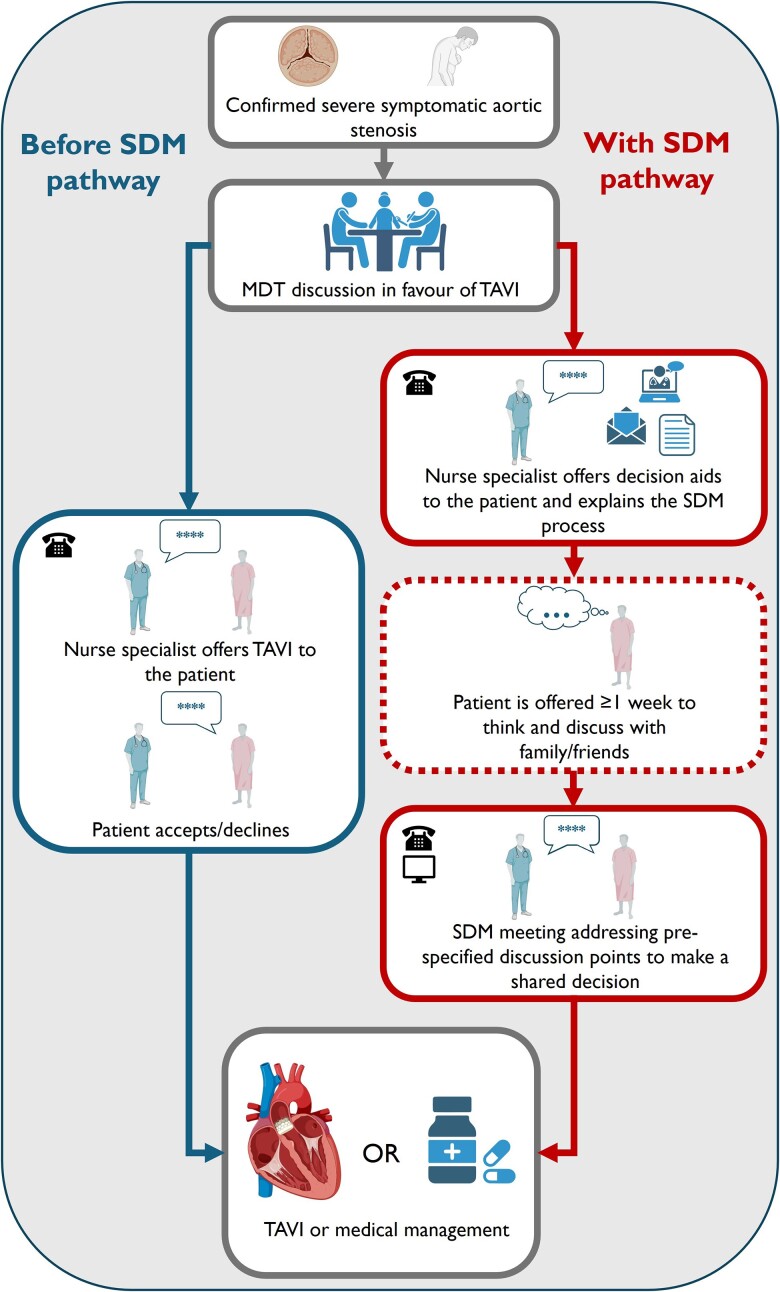
Flow chart depicting the changes in our local practice before (left) and after (right) introducing a formal shared decision-making pathway for transcatheter aortic valve implantation. Before the introduction of the shared decision-making pathway, the patient was communicated the opinion of the multi-disciplinary team by phone and normally, after a discussion with the nurse, decided whether to accept or not. With the shared decision-making pathway, patients are now offered 1 week to visualize the information provided via email/post, talk to their family and/or friends, and build an informed opinion. This permits active participation in the shared decision-making meeting, normally held remotely, where the nurse recapitulates all the details of the aortic stenosis and potential treatment options, to make a shared decision on how to proceed. Image created with the help of BioRender.com.

Before the introduction of the SDM pathway, a decision was made during the multi-disciplinary team (MDT) meeting whether to offer TAVI, surgery, or medical management, and consequently the patient was contacted and offered the treatment (*Figure 2*). If the patient accepted the TAVI, there was a pre-admission meeting followed by admission for the procedure.

The key difference in the pathway is that now the patient is communicated the MDT opinion to recommend TAVI, but not immediately offered the treatment. Instead, our staff (normally a clinical nurse specialist) explains that the ultimate decision on the treatment will be made in agreement between the patient and the clinical team, once the patient is fully aware of the reasons behind the treatment choice and the other potential options, and after consulting with their family and/or friends if they wish to. The patient receives decision aid leaflets and a video with more details on the procedure via email or alternatively via post (with an URL for the video) and is then contacted again after a week for the formal SDM meeting. During the meeting, which is normally held remotely by telephone and lasts ∼30 min, the following aspects are covered:

- Aortic stenosis as a medical condition, including the usual progression of the disease, aortic stenosis disease severity definition, its relationship to symptoms, when treatment might be indicated in accordance with the international guidelines, the relationship between the valve narrowing and symptoms, and how this affects recommended treatment.
- Management options for aortic stenosis, including regular monitoring (watch and wait), open heart surgery, TAVI, or referral to palliative care for help to optimize quality of life, with the risks and likely outcomes of each management approach.
- Patient’s values, circumstances, and preferences, which are specifically sought and addressed as part of the meeting.

At the end of the meeting, the patient expresses their final decision, or they can ask to be contacted again in 1–2 weeks if they would like more time. Minutes are then taken from the SDM meeting and published on the institutional electronic patient record.

### Evaluation questionnaire

To verify whether the new TAVI pathway was effective in complying with the NICE and PRSB standards, alongside the patient’s understanding of the treatment options and satisfaction with the way the decision process was managed, we commissioned the design of a dedicated post-operative questionnaire. The questionnaire (developed by Civica Group Limited) was designed across selected domains, modifying the already existing SDM-Q-9 to specifically address the NICE and PRSB standards,^9,13,20^ and administered via an online tool. The questionnaire was available both in virtual and paper versions.

We started administering the questionnaire to patients before the SDM pathway was implemented and then continued after the implementation of the pathway, to look for differences in the global satisfaction between the pre-SDM and post-SDM patients, along with individual question answers. Global satisfaction is the standard measure adopted in questionnaires assessing SDM and was therefore deemed to be the most reliable outcome.^13–18^ Patient demographics were also investigated in the questionnaire to ensure that no obvious contextual elements could be a source of bias.

## Methods

This manuscript has been designed in compliance with the Standards for QUality Improvement Reporting Excellence 2.0 guidelines.^21^

The questionnaire consisted of 30 questions including details on the demographics, admission route, 2 free-text questions, and a core of 12 questions (*Table 1*) graded with a Likert scale and based on NICE guidance, PRSB criteria, and SDM-Q-9.^9,13,20^ Points were assigned from 1 to 5 with 1 representing strong disagreement and 5 representing strong agreement.

**Table 1:** Core questionnaire questions

Core questionnaire questions
1. My doctor and I reached an agreement on how to proceed
2. My doctor and I selected a treatment option together
3. My doctor and I thoroughly assessed the different treatment options
4. My doctor asked exactly how I want to be involved in making the decision
5. My doctor asked me which treatment option I preferred
6. My doctor explained the advantages and disadvantages of the treatment options
7. My doctor gave me enough information to understand the different treatment options
8. My doctor gave me enough time to consider the treatment options
9. My doctor gave me the opportunity to ask questions about the treatment options
10. My doctor helped me understand all the information provided
11. My doctor told me that a decision about my treatment and care needs to be made
12. My doctor told me that there are different options for treating my medical condition

The questionnaire was designed across selected domains, modifying the already existing SDM-Q-9 to specifically address the NICE and PRSB standards.

The questionnaire was administered via an online tool to a cohort of 23 post-TAVI patients enrolled between 22 November 2022 and 14 January 2023 (before the implementation of the SDM programme), and 38 patients enrolled between 15 January 2023 and 30 June 2023 (after the implementation of the SDM programme).

### Statistical analysis

To investigate differences in the global satisfaction between the two patient groups (pre-SDM vs. post-SDM), we compared them based on the Likert scale values assigned to the 12 core questionnaire questions using the Wilcoxon rank sum test with continuity correction; α was set at 0.05%.

We computed the global satisfaction score (GSS) as the mean of the raw scores from all questions answered by the patients in the individual group and selected the standard error of the mean as measure of uncertainty. Differences in the demographic characteristics (excluding age), in the number of patients who responded positively (Likert 4–5) or negatively (Likert 1–2) to each individual question, and in the percentages of positive and negative answers between pre-SDM and post-SDM were analysed with χ^2^ test or Fisher exact test where at least one of the expected values was <5. For age, Spearman’s rank correlation was used. The answer ‘prefer not to say’ was excluded from the analysis. The statistical analysis was performed with RStudio 2022.12.0 Build 353.

### Patient and public involvement statement

Data presented are completely anonymized; therefore, no patient consent was sought for this publication.

This research was conducted as part of a routine institutional audit with the aim of improving the SDM process and assessing patient awareness and satisfaction. Our questionnaire was developed from SDM-Q-9,^13^ which was in turn designed with the contribution of patients, and adapted to incorporate the standards in the NICE SDM guidance.^9^ In addition, we introduced a specific free-text question (‘Please use this space if there is anything else you want to tell us about your involvement in making decisions with your clinician about your treatment and care.’) to collect the opinions of patients as expressed in their own words.

## Results

After excluding patients who participated in the questionnaire but did not answer any of the 12 Likert scale questions, the remaining population accounted for 20 pre-SDM and 27 post-SDM patients. Contextual elements that contributed to the outcomes were assessed with questions about demographics, equity, and diversity, and no significant differences were found between the groups (*Table 2*).

**Table 2:** Patient demographics questions

What do you consider to be your ethnic background?				In which age group are you?			
Available responses	Without SDM pathway	With SDM pathway	*P*	Available responses	Without SDM pathway	With SDM pathway	*P*
White British	85% (17)	85.19% (23)	0.300	Under 16 years	0% (0)	0% (0)	0.492
White Irish	5% (1)	3.70% (1)		16–24	0% (0)	0% (0)	
Any other White background	0% (0)	7.41% (2)		25–34	0% (0)	0% (0)	
Black Caribbean	0% (0)	0% (0)		35–44	0% (0)	0% (0)	
Black African	0% (0)	0% (0)		45–54	5% (1)	0% (0)	
Any other Black background	0% (0)	0% (0)		55–64	0% (0)	11.11% (3)	
Bangladeshi	0% (0)	0% (0)		65–74	25% (5)	3.70% (1)	
Chinese	0% (0)	0% (0)		75–84	50% (10)	40.74% (11)	
Indian	0% (0)	0% (0)		85+	20% (4)	40.74% (11)	
Pakistani	0% (0)	0% (0)		Did not answer	0% (0)	3.70% (1)	
Any other Asian background	5% (1)	0% (0)		Do you have any of the following physical or mental health conditions or disabilities?			
White and Black Caribbean	0% (0)	0% (0)		Available responses	Without SDM pathway	With SDM pathway	*P*
White and Black African	0% (0)	0% (0)		Mobility difficulty	40% (8)	29.63% (8)	1
White and Asian	5% (1)	0% (0)		Blindness or partial sight	5% (1)	0% (0)	
Any other mixed/multiple ethnic background	0% (0)	0% (0)		Deafness or hearing loss	15% (3)	22.22% (6)	
Other	0% (0)	0% (0)		Communication difficulty	0% (0)	0% (0)	
Prefer not to say	0% (0)	0% (0)		Learning disability	0% (0)	0% (0)	
Did not answer	0% (0)	3.70% (1)		Mental health condition	0% (0)	0% (0)	
What is your gender?				Other	10% (2)	3.70% (1)	
Available responses	Without SDM pathway	With SDM pathway	*P*	I do not have a disability	20% (4)	29.63% (8)	
Male	55% (11)	62.96% (17)	0.474	Prefer not to say	5% (1)	7.41% (2)	
Female	45% (9)	33.33% (9)		Did not answer	5% (1)	7.41% (2)	
Did not answer	0% (0)	3.70% (1)					

This table reports the answers to questions regarding patient gender, age, religion, ethnicity, and disability.

**Figure 3:**
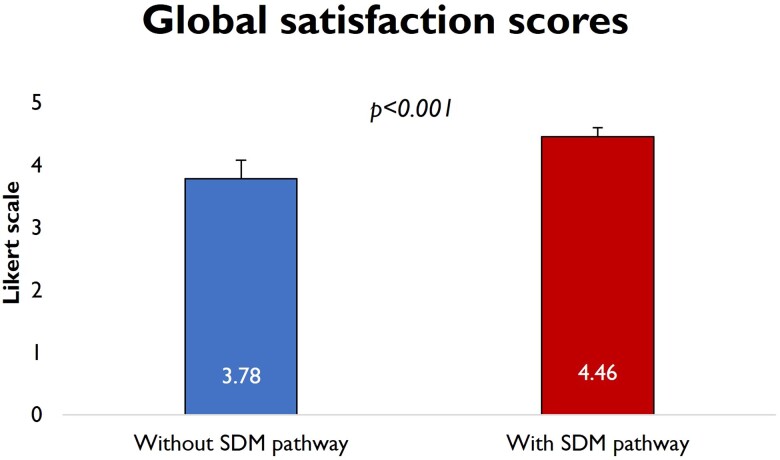
Global satisfaction scores. There is a statistically significant difference in the global satisfaction between patients who went through the shared decision-making pathway and those who did not (global satisfaction score 4.46 ± 0.14 vs. 3.78 ± 0.30, *P* < 0.001).

After the implementation of the SDM pathway, there was a significant improvement in the global satisfaction (3.78 ± 0.30 in the pre-SDM group vs. 4.46 ± 0.14 in the post-SDM group, *P* < 0.001; *Figure 3*). The percentage of positive (Likert 4–5) responses was significantly higher in the post-SDM group (155/234, 66.2% pre-SDM vs. 289/312, 92.6% post-SDM, χ^2^ = 61.3, *P* < 0.001), whereas the percentage of negative responses was significantly lower in the post-SDM group (53/234, 22.6% pre-SDM vs. 5/312, 1.6% post-SDM, χ^2^ = 62.4, *P* < 0.001).

**Figure 4:**
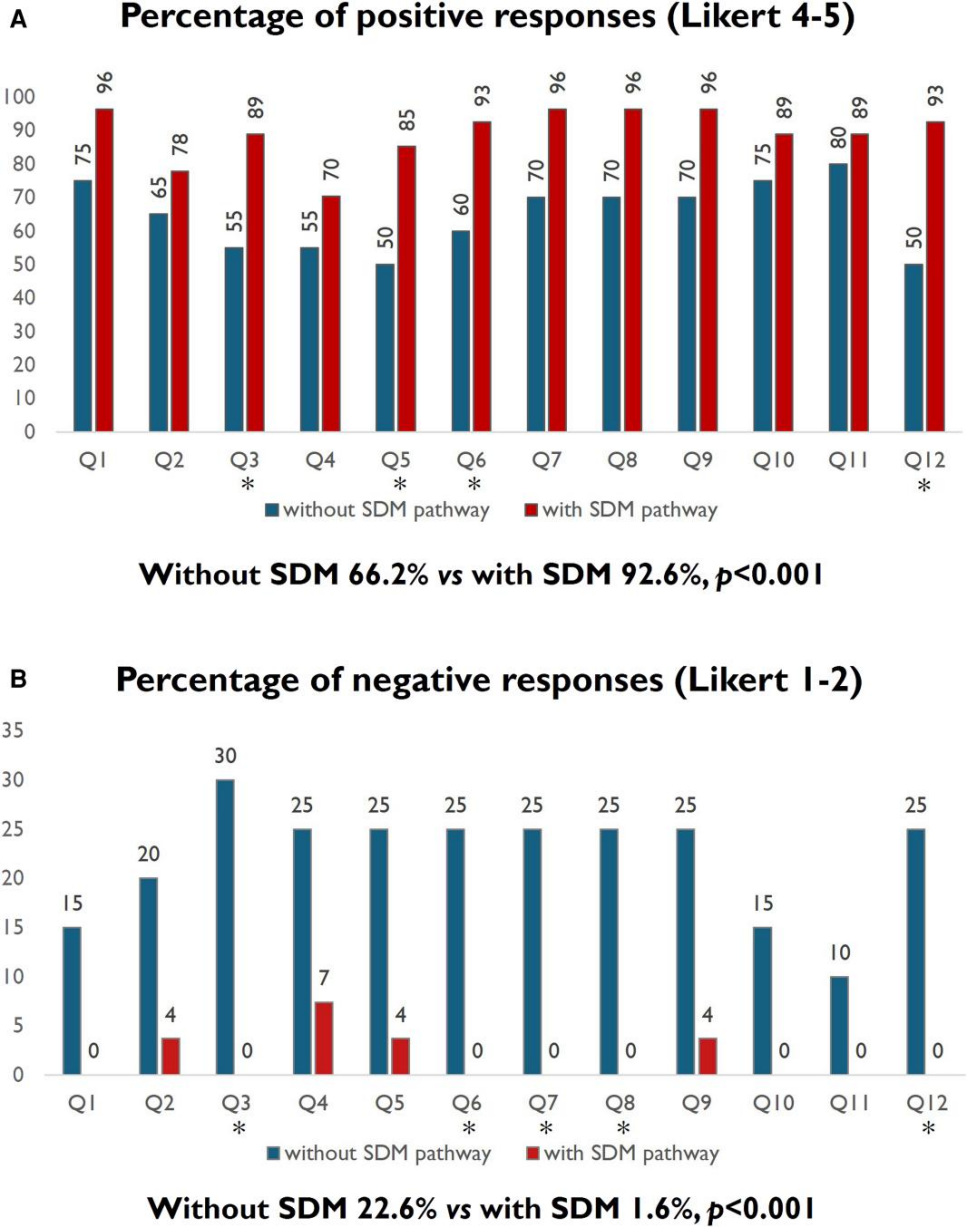
Percentages of positive and negative responses to specific questions. For each couple of bars, left is without shared decision-making pathway and right is with shared decision-making pathway. Questions answered positively/negatively by significantly different percentage of patients are highlighted with a symbol ‘*’. (*A*) The percentage of patients who were engaged in shared decision-making and responded positively (Likert 4–5) exhibited a higher trend for each question, and four questions reached statistical significance (Q3 *P* = 0.0161, Q5 *P* = 0.0123, Q6 *P* = 0.0111, and Q12 *P* = 0.0017). The overall percentage of positive responses was significantly higher in the post-shared decision-making group (155/234, 66.2% vs. 289/312, 92.6%, *P* < 0.001). (*B*) The percentage of patients who were engaged in shared decision-making and responded negatively (Likert 1–2) exhibited a lower trend for each question, and five questions reached statistical significance (Q3 *P* = 0.0036, Q6 *P* = 0.0101, Q7 *P* = 0.0101, Q8 *P* = 0.0101, and Q12 *P* = 0.0101). Only a small proportion of patients in the shared decision-making pathway gave negative answers (4% for Q2, Q5, and Q9, and 7% for Q4). The overall percentage of negative responses was significantly lower in the post-shared decision-making group (53/234, 22.6% vs. 5/312, 1.6%, *P* < 0.001).

For all questions, the percentage of post-SDM patients who responded positively exhibited a higher trend than pre-SDM (*Figure 4*). The individual questions with a statistically significant higher number of positive answers were no. 12 [‘My doctor told me that there are different options for treating my medical condition.’ (50 vs. 93%, *P* = 0.0017)], no. 6 [‘My doctor explained the advantages and disadvantages of the treatment options.’ (93 vs. 60%, *P* = 0.0111)], no. 5 [‘My doctor asked me which treatment option I preferred.’ (50 vs. 85%, *P* = 0.0123)], and no. 3 [‘My doctor and I thoroughly assessed the different treatment options.’ (55 vs. 89%, *P* = 0.0161)].

For all the questions, the rate of negative answers was lower in both patient groups (see *Figure 4* for statistically significant questions). In the pre-SDM group, they ranged 10–30%, while in the post-SDM group, they were almost nil: only three questions (no. 2 ‘My doctor and I selected a treatment option together.’, no. 5 ‘My doctor asked me which treatment option I preferred.’, and no. 9 ‘My doctor gave me the opportunity to ask questions about the treatment options.’) received a single negative answer (4%), and one question (no. 4 ‘My doctor asked exactly how I want to be involved in making the decision.’) received two negative answers (7%).

Concerning the free-text question on patient involvement in SDM:

- For the pre-SDM group, 9/20 (45%) entered free text. In summary, six were very positive about the process. One patient was concerned that through cancellation they had no face-to-face contact until late in the process, one patient was concerned that the process was unclear until they were telephoned and offered TAVI, and one patient’s family reported that they underwent post-procedural delirium, and they were not sure that the patient would have wanted the TAVI if they had been aware of this risk. For these 3 patients, the average Likert score in the 12 core questions was 2.08, 2.00, and 4.58, respectively (pre-SDM group GSS: 3.78 ± 0.30).
- For the post-SDM group, 6/27 (22%) filled in the free-text field. Four patients gave very positive responses and two patients felt that they were given too much information with the decision aids. For these two patients, the average Likert score was 4.75 and 3.18 (post-SDM group GSS: 4.44 ± 0.14).

## Discussion

In parallel with the progressive technological development of complex medical treatments, there is an increasing awareness of the importance of informed patient engagement in clinical decision-making. Indeed, technological developments have allowed treatments such as TAVI to become feasible for patients who would have been too frail for conventional surgery. This represents both an opportunity for patient benefit and a risk of potentially causing harm or a less good outcome than conservative therapy, underpinning the need for SDM.

All parties benefit from SDM. The patient is fully aware of all the options and the risks of the chosen procedure, tends to be more compliant with treatment and medications, and ultimately feels more satisfied with the service received. The clinical team can work in a more collaborative way with a fully aware patient; the healthcare facility and system are less likely to receive complaints and cancellations due to second thoughts. The evidence of the benefits of SDM comes from multiple clinical studies in cardiovascular disease.^22–27^

In this manuscript, we have presented the results of our experience with the implementation of a structured SDM pathway, which has led to a significant global satisfaction improvement based on the post-procedural questionnaire. Our questionnaire was specifically designed to verify our effectiveness in informing and engaging the patient based on the NICE and PRSB standards, which improved significantly with the introduction of a structured SDM pathway. Based on the optimized design of our questionnaire and the lack of statistically significant differences in the baseline patient characteristics, the most likely reason for the observed improvement in global satisfaction is the introduction of the SDM pathway. The percentage of negative answers provided by pre-SDM patients was already low at 10–30%, reflecting the fact that before establishing the SDM pathway there was a good degree of satisfaction about discussion of the treatment alternatives and patient preferences. However, the negative answers were almost nil in the post-SDM group. Moreover, the statistically significant differences in positive and negative answers suggest a substantial improvement in communication with the patient and in patient’s awareness and engagement in the treatment decision, particularly through joint assessment of the different treatment options including advantages, disadvantages, and general information, involvement of the patient in expressing a preference, and appropriate amount of time to consider the treatment options.

The strengths of our work are the reliance on a guideline-based approach to SDM and the design of a dedicated questionnaire to measure the performance of our SDM pathway. We also acknowledge some limitations, including a small, non-randomized, and single-centre patient population, the absence of blinding, and the difficulty of incorporating additional useful outcomes such as resource utilization, costs, length of stay, compliance, or patient anxiety in our protocol, which was based on a simple opinion-based questionnaire. Moreover, we kept demographic and clinical information on the patients to a minimum, to maximize the ease of completion.

However, we believe that these limitations are counterbalanced by the optimized design of our questionnaire to assess guideline-driven outcomes.

Our results add to the evidence base that SDM is effective in terms of patient understanding of their condition and different treatment options, and satisfaction in the whole decision-making process. This is particularly the case for procedures like TAVI where there are treatment alternatives that may suit the patient’s needs better. Our questionnaire proved a useful tool to assess the efficacy of SDM.

There is extensive literature supporting the notion that SDM tools may increase patient knowledge, engagement, and satisfaction.^28–31^ Moreover, there is evidence that SDM may reduce healthcare-associated costs^11,31,32^ across different settings, including hip and knee surgery,^33^ children with special healthcare needs,^34^ or inflammatory bowel diseases.^35^ Overall, the reduction in costs seems to be mainly driven by a lower number of procedures performed when the patient is actively involved in the decision-making. Interestingly, this means that a significant proportion of symptom-driven interventions might be performed without a real benefit for patients who, if adequately informed and involved, would opt for conservative management. Other contributors to cost reduction have been found to be decreased rates of hospitalizations, emergency department visits, and office visits. This is likely the result of the patient’s better awareness of the disease symptoms and course, which might explain a reduced need for consultations.

An SDM pathway such as the one we implemented is relatively inexpensive and can be carried out by a trained nurse specialist. Moreover, the use of digital technology, including online information aids and remote meetings/consultations, may further reduces costs and increases patient convenience. A similar model could be easily applied to other clinical contexts such as chronic coronary artery disease management^36^ or decision for left ventricular assist device/heart transplantation in advanced heart failure,^37^ where the treatment can be either conservative or interventional/surgical and should generally be guided by a combination of symptoms, prognostic implications, and most importantly patient choice. For example, following an MDT discussion on a complex case of multivessel coronary artery disease, a clinical specialist nurse could approach the patient telephonically to communicate the outcome of the MDT, which was in favour of offering the patient bypass surgery. The nurse could then offer information aids and videos explaining the different treatment options (conservative management, percutaneous intervention, or surgery) and set up a meeting for 1 week later. The patient would have time to reflect and consult their family or friends, and after 1 week there would be a formal SDM meeting, where the nurse recapitulates the implications of the disease and treatment options and initiates a discussion to reach a shared decision with the patient. This process would grant the patient a better knowledge and empower them to make an informed decision. When properly informed, some patients might simply prefer medical management over an invasive procedure, which would be both cost-saving and in line with the patient’s desires. Patients who would opt for an invasive procedure would be fully aware of the implications and most likely be more satisfied with the overall process and less prone to cancel the procedure.

## Conclusions

The results of our post-procedural questionnaire suggest that the structured TAVI SDM pathway is effective in giving patients the tools to make an informed decision in collaboration with the clinical team, with a high level of satisfaction. The wider roll out of SDM with digital technologies into clinical pathways has the potential to significantly improve patient understanding, awareness, and overall satisfaction.

## Data Availability

Spreadsheets with the anonymized questionnaire results are available upon reasonable request. Please contact Dr Ermes Carulli at e.carulli@rbht.nhs.uk. Reuse is not permitted unless otherwise agreed.
